# Supplementary material for: MetaRibo-Seq measures translation in microbiomes
Source: Nat Commun. 2020 Jun 29;11:3268. doi: 10.1038/s41467-020-17081-z (PMC7324362; doi:10.1038/s41467-020-17081-z)
Supplement: Supplementary file 10 — Supplementary Data 7 [file 41467_2020_17081_MOESM10_ESM.zip › File2/Confidence_High_Taxonomy/242480_out.krona.html]

Javascript must be enabled to view this page.

members
magnitude
magnitudeUnassigned
count
unassigned
taxon
rank

242480\_out

5

2
superkingdom
5

1239
5
phylum

186801
5
class

2
5
186802

SRS053356\_contig\_number\_45537SRS098571\_contig\_number\_contig-100\_2100.370630
order

541000
3
family

genus
3
946234

3
species

SRS013687\_contig\_number\_contig-100\_5295.158738SRS051031\_contig\_number\_34835SRS1041145\_contig\_number\_39348
1193534
